# Quadrilateral Space Syndrome: Diagnosis and Clinical Management

**DOI:** 10.3390/jcm7040086

**Published:** 2018-04-21

**Authors:** Patrick T. Hangge, Ilana Breen, Hassan Albadawi, M. Grace Knuttinen, Sailendra G. Naidu, Rahmi Oklu

**Affiliations:** 1 Department of General Surgery, Mayo Clinic, Phoenix, AZ 85054, USA; 2 Department of Vascular Interventional Surgery, Minimally Invasive Therapeutics Laboratory, Mayo Clinic, Phoenix, AZ 85054, USA; breen.ilana@mayo.edu (I.B.); albadawi.hassan@mayo.edu (H.A.); Knuttinen.Grace@mayo.edu (M.G.K.); naidu.sailen@mayo.edu (S.G.N.); 3 Mayo Clinic School of Medicine, Scottsdale, AZ 85259, USA

**Keywords:** quadrilateral space syndrome, axillary nerve, posterior humeral circumflex artery, magnetic resonance imaging, digital subtraction angiography

## Abstract

Quadrilateral space syndrome (QSS) is a rare disorder characterized by axillary nerve and posterior humeral circumflex artery (PHCA) compression within the quadrilateral space. Impingement is most frequently due to trauma, fibrous bands, or hypertrophy of one of the muscular borders. Diagnosis can be complicated by the presence of concurrent traumatic injuries, particularly in athletes. Since many other conditions can mimic QSS, it is often a diagnosis of exclusion. Conservative treatment is often first trialed, including physical exercise modification, physical therapy, and therapeutic massage. In patients unrelieved by conservative measures, surgical decompression of the quadrilateral space may be indicated.

## 1. Introduction

Quadrilateral space syndrome (QSS) is a rare disorder characterized by axillary nerve and posterior humeral circumflex artery (PHCA) compression within the quadrilateral space, first described by Cahill and Palmer in 1983 [1,2,3,4,5]. Cahill originally described four distinct features of QSS: (1) diffuse pain around the shoulder; (2) paresthesia in a nondermatomal distribution; (3) point tenderness above the quadrilateral space; and (4) positive angiogram finding in provocative positioning [5]. The condition is commonly attributed to repeated overhead activity (such as baseball or volleyball athletes), but a variety of other pathologies including lipomas, hematomas, and labral cysts may cause compression in the quadrilateral space [6]. 

While neurovascular compression does account for the acute findings in QSS including pain, paraesthesia, and atrophy, it is debated whether this is primarily a problem of neural entrapment or vascular compression [7,8]. Compression may occur at rest or with movement and QSS should be considered for all patients with a chief complaint of neck pain, shoulder pain, lateral arm paraesthesia, and/or quadrilateral space tenderness [3].

## 2. Anatomy

**Figure 1:**
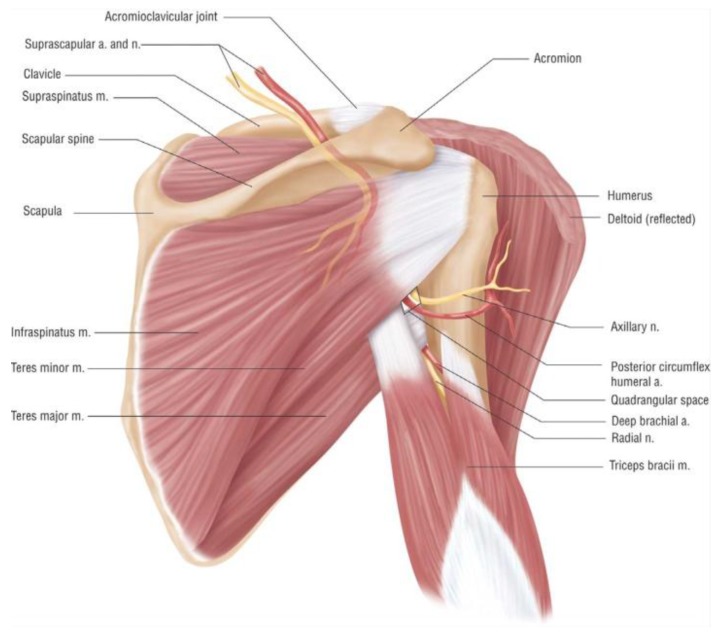
Anatomy of the quadrilateral space. This posterior view of the right shoulder shows the quadrilateral space which is bounded superiorly by the teres minor muscle, inferiorly by the teres major muscle, medially by the long head of the triceps, and laterally by the humeral shaft (reprinted with permission from [14].

The quadrilateral space is bounded superiorly by the teres minor muscle, inferiorly by the teres major muscle, medially by the long head of the triceps, and laterally by the humeral shaft [7] (Figure 1). The axillary nerve and PHCA reside in the quadrilateral space. The axillary nerve innervates the teres minor and deltoid muscles, which are primarily responsible for abduction and external rotation. Characteristic fibrous bands are found within the quadrilateral space, which exacerbate symptoms, particularly pain, elicited by movements associated with the deltoid and teres minor muscles [9,10]. The anatomical differences in innervation patterns in the glenohumeral joint between patients can make it difficult to distinguish whether pain is due to suprascapular nerve palsy or axillary nerve compression [11]. Because the PHCA stretches around the neck of the humerus, repetitive tension and mechanical stress to the PHCA wall can lead to thrombosis and aneurysmal degeneration [4,12,13].

## 3. Etiology

The etiology of QSS is unclear, but impingement is most frequently due to trauma, fibrous bands, or hypertrophy of a muscular border. In rare cases, QSS has also been caused by labral cysts, hematoma resulting from fracture, osteochondroma, lipomas, and axillary schwannomas. Compression of the axillary nerve can also follow aneurysms and traumatic pseudoaneurysms of the posterior circumflex humoral artery [13,15,16,17]. Additionally, anatomical variations can predispose patients to QSS. For example, abnormal origin of the radial collateral artery from the PHCA, however rare, can also mimic the symptoms of QSS [18]. In another case, the discovery of an accessory subscapularis muscle that originates from the anterior surface of the subscapularis, courses under the axillary nerve, and inserts onto the shoulder joint, can also serve as a rare risk factor for QSS [19]. QSS has also been described as a rare complication of thoracic surgery [20].

## 4. Presentation, Differential Diagnoses, Imaging, and Other Workup

### 4.1. Presentation

Reported cases of QSS typically involve younger patients, less than forty years of age, who are otherwise healthy. Patients can present with a history of repeated overhead activities, for example, in athletes of volleyball, baseball, or swimming [21,22,23,24,25,26,27,28]. Symptom presentation can be vague with involvement of neurogenic or vascular features. Neurogenic QSS is characterized by paresthesia, fasciculations, weakness, or neurogenic pain in a nonspecific pattern. Symptoms suggestive of vascular QSS include signs of acute ischemia (pain, pallor, absent pulses), thrombosis, or embolism (coolness or cyanosis of the hand or digits). In addition to vascular and neurogenic symptoms, patients with QSS can experience muscular atrophy and accompanying weakness, thought to be a result of denervation. Patients can also present with tenderness over the quadrilateral space. In severe cases, thrombosis of the PHCA can block flow from the axillary artery, causing embolization and subsequent cyanosis, digital ischemia, and cold intolerance [4,29,30]. 

### 4.2. Differential Diagnoses

Nondescript symptoms often make QSS a diagnosis of exclusion as it can mimic other musculoskeletal, vascular, or nerve-related syndromes in the area. A review of the patient’s medical history may also be useful; particularly the absence of significant relief following attempted therapeutic interventions may be noted. One patient underwent the following procedures before a diagnosis of QSS in our clinic: labral repair, subacromial decompression, exploratory surgery, first rib resection, anterior/middle scalenectomy, neurolysis, and pectoralis minor release. This patient also attempted acupuncture, extensive physical therapy, and steroid injections.

Diagnoses to rule out in the workup of QSS include rotator cuff injuries, referred pain syndromes, cervical spine pathologies, and labral injuries [3]. Other important conditions that should be considered in the differential include brachial plexus pathologies, such as thoracic outlet syndrome and brachial neuritis, glenohumeral joint arthritis, and suprascapular nerve injury [7]. Fracture of the head of the humerus, anterior shoulder dislocation, and blunt trauma can all be sources of axillary nerve injury independent of axillary nerve compression. In one report, a patient presenting with QSS symptoms (shoulder pain and upper limb numbness when throwing) was ultimately found to have compression of the axillary nerve between the proximal humerus and the latissimus dorsi tendon [21]. In this situation, the clinical sequelae can be identical, but the nerve compression occurs outside of the quadrilateral space. 

### 4.3. Imaging and Other Workup

In the workup of QSS, given the often vague patient presentation, imaging is important to both rule out and rule in QSS. Imaging of QSS can be challenging since compression of the axillary nerve may be intermittent, frequently due to positional dependence. Holding the arm in flexion, abduction, and external rotation for several minutes has been described as an effective method to reproduce symptoms and secure the diagnosis [25]. When QSS is suspected, the diagnosis is often confirmed by imaging [3]. 

**Figure 2:**
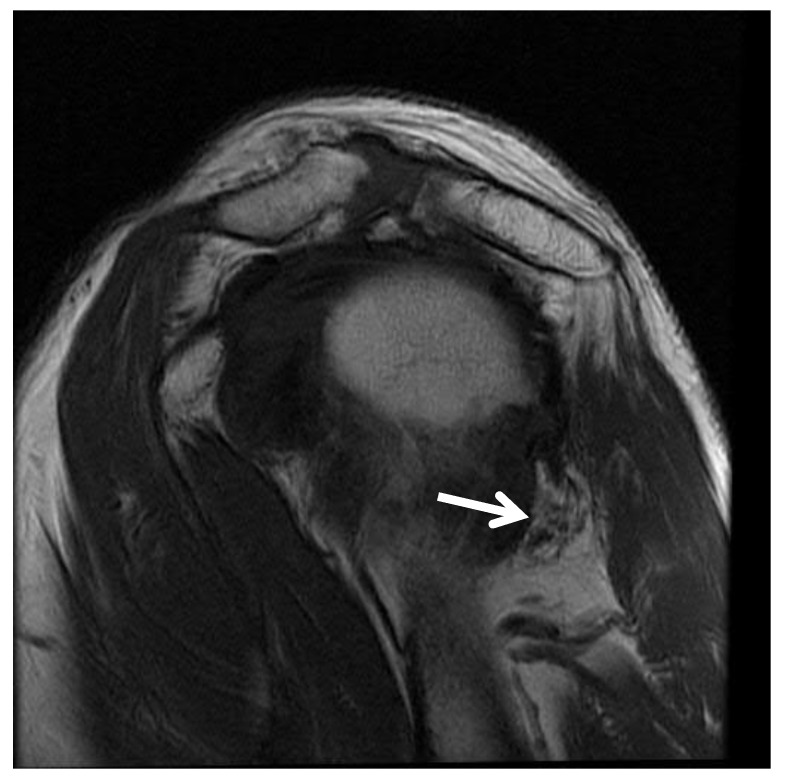
Atrophic left teres minor on magnetic resonance imaging (MRI) of shoulder in patient with quadrilateral space syndrome.

**Figure 3:**
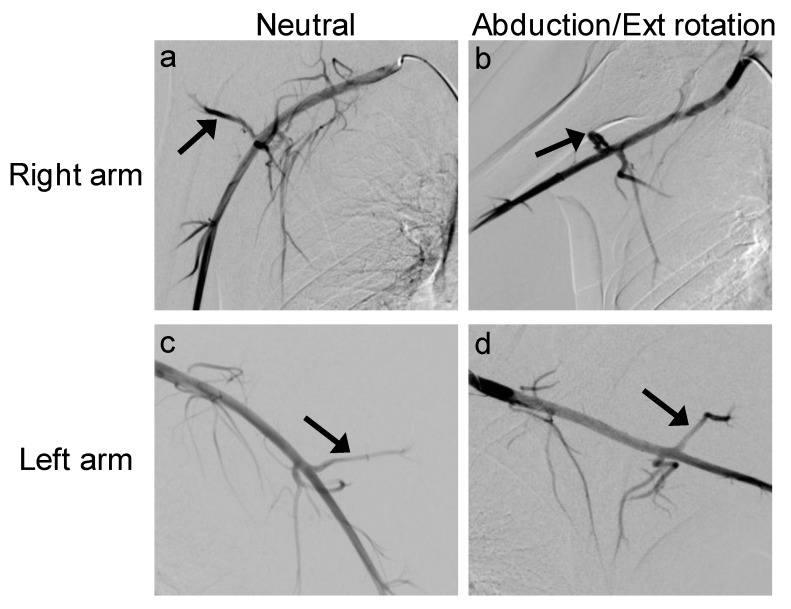
A 27-year-old right hand-dominant man presented with right shoulder pain and weakness, which had been nonresponsive to conservative measures. His pain was located in the right posterior shoulder with point tenderness directly over the quadrilateral space. This pain was aggravated by abduction and external and internal rotation. Following clinical examination, bilateral upper extremity arteriography was performed in neutral position and following provocative maneuvers. Bilateral provocative digital subtraction angiography of posterior humeral circumflex artery (PHCA) in patient with chronic right-sided shoulder pain. (**a**) Arrow pointing to normal flow of right PHCA with patient in neutral, supine position. (**b**) Arrow pointing to external compression of right PHCA following abduction and external (Ext) rotation of right shoulder. (**c**) Arrow pointing to normal flow of left PHCA with patient in neural, supine position. (**d**) Arrow pointing to normal flow of left PHCA following abduction and external rotation of left shoulder.

Digital subtraction angiography, computed tomography angiography, and magnetic resonance angiography have all been used to visualize PHCA occlusion [2,31]. While there is no “gold standard” diagnostic test for QSS, magnetic resonance imaging (MRI) is typically the first choice of imaging [32]. MRI often demonstrates focal fatty atrophy of the teres minor muscle and can exclude pathological causes of shoulder pain (Figure 2) [1,7,32]. Arteriography has been described as the cornerstone of diagnosis in QSS; it is used to reveal compression of the PHCA while the patient’s arm is in abduction and external rotation [3] (Figure 3). Bilateral upper extremity arteriography is useful in establishing the patient’s baseline healthy anatomy, as compared to the pathological shoulder. Decreased outflow from the PHCA would indicate compression of the adjacent axillary nerve leading to QSS [3,7]. However, in one controlled study, 80% of asymptomatic controls demonstrated PHCA occlusion in arteriography, leading to low specificity [31]. 

A recent report described ultrasound diagnosis which demonstrated a dilated PHCA and mild atrophy of the deltoid muscle on the affected side [33]. In another report, occlusion and stenosis of PHCA were detected using ultrasound [17]. Although less common, teres minor atrophy can also be seen [34,35,36]. Color Doppler sonography has also been used to compare differences in the posterior humeral circumflex arterial flow between neutral and provocative positions [37]. Sonoelastography has also been used in the diagnosis of QSS secondary to axillary schwannoma [38]. While no data exists regarding its superiority over traditional imaging methods using angiography, ultrasound offers a readily available, cheaper option to investigate QSS.

Others have described the potential usefulness of the electromyogram (EMG). EMG can detect denervation of muscles supplied by a compressed axillary nerve, such as the teres minor and deltoid muscles. However, the test has a high false-negative rate. In one study, four patients with established QSS underwent EMG and MRI testing. All four EMG scans were negative and half of the MRI scans were normal [39]. While EMG can yield false negative results, it can still eliminate other etiologies of neuropathic pain, such as neurogenic thoracic outlet syndrome. 

## 5. Treatment

There is great variability in the management and treatment of QSS in the literature, due in part to its recent description and because case reports dominate the literature. Typically, conservative measures such as physical therapy and physical activity modification are first recommended to patients [22]. Physical activity modification can be useful in the initial treatment of QSS; however, some patients may find this hard to do and there may be reluctance on behalf of the patients. Another conservative therapeutic option, physical therapy, can include transverse friction massage and active release soft tissue massage techniques to the quadrilateral space [3]. In addition to therapeutic massage, active shoulder range of motion and scapular stabilization exercises, stretching of the posterior rotator cuff, and the use of nonsteroidal anti-inflammatories have shown success in managing QSS [3]. Another conservative measure met with mild success in the literature is the use of ultrasound-guided perineural steroid injections. Pain and other symptomatic relief (tingling, sensation of coldness) following the injection of local anesthetic or steroids can be a diagnostic sign for QSS, but can also be used with physical therapy for symptomatic management [6]. 

Surgical decompression is considered when patients are unresponsive to conservative measures for at least six months [40]. Decompression has proven successful in reversing radial sensory neuropathy secondary to QSS [29]. Since other conditions can mimic QSS, including effort thrombosis (also known as Paget–Schroetter syndrome) and arterial thoracic outlet syndrome, imaging and pertinent follow-up testing is essential before planning interventional decompression [41,42]. Despite its lack of specificity, arteriography demonstrating compression of the PHCA and the accompanying presentation of classic QSS symptoms should raise a high index of suspicion for QSS and surgical decompression may be indicated. In surgical decompression, the axillary nerve is dissected free to ensure competency. During the procedure, the axillary nerve and PHCA can be palpated while the patient’s arm is placed in external rotation and abduction, to verify a freely gliding uncompressed nerve and a consistently strong pulse in the artery [43]. It is also useful to check for fibrous bands around the neurovasculature, which may be indicative of structural compression [39]. Postoperatively, patients can be placed in an arm sling for comfort with immediate physical therapy to avoid the development of adhesions. Physical therapy should be a central part to the postoperative recovery [39]. Following successful operation, most athlete patients can return to their sport [22].

There are several other treatment modalities reported in literature, including thrombolysis in the setting of thrombus, thrombectomy in the setting of distal emboli formation, and aneurysm resection and endovascular treatment with coiling [44,45,46,47]. In rare instances, quadrilateral space syndrome may spontaneously resolve [48].

## 6. Conclusions

QSS is a rare disorder with signficant morbidity. Because many symptoms of QSS are nonspecific, there may be a delay in diagnosis and subsequent delay in care. In one study, it was shown that the mean interval from the commencement of symptoms to surgical decompression was 14.5 months, with a range from 6–24 months [39]. Practitioner awareness of QSS as a differential diagnosis, particularly in at-risk populations such as athletes, is also critical for timely and accurate diagnosis. Careful analysis of imaging studies is necessary to exclude differential diagnoses, understand etiology, and to devise the best treatment strategy for QSS. Future investigations could include studies on the prevalence of QSS and a more thorough analysis on the barriers of diagnosis, including limited practitioner awareness of the condition, nonspecific presentation, and the absence of a gold standard workup.
